# Supplementary figures and images for: Liposomal Dexamethasone Reduces A/H1N1 Influenza-Associated Morbidity in Mice
Source: Front Microbiol. 2022 Apr 12;13:845795. doi: 10.3389/fmicb.2022.845795 (PMC9048800; doi:10.3389/fmicb.2022.845795)

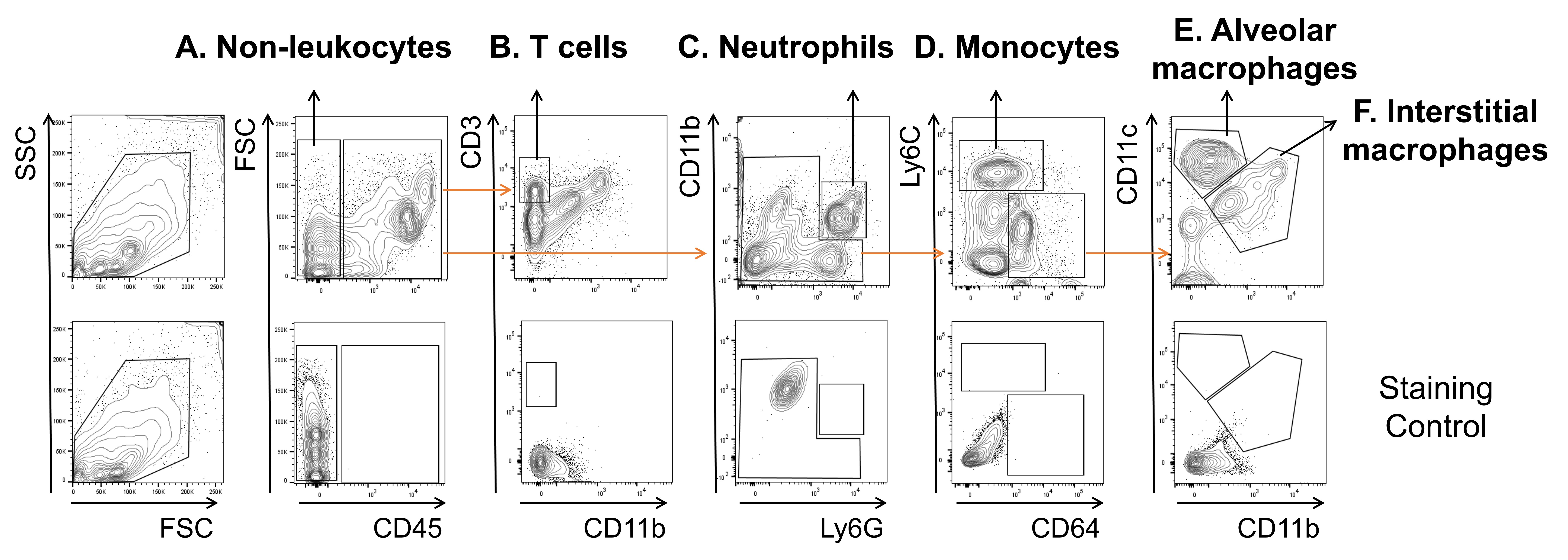

Supplement: Supplementary Figure 1 — Gating strategies to determine the cellular compositions of the lungs of mice (Related to Figure 1). Single cells were isolated from the lungs of DiI+ injected mice and analyzed by flow cytometry. Sequentially gated cells were (A) CD-45– non-leukocytes, (B) CD45+ CD11b–CD3+ T cells, (C) CD45+ CD11b+Ly6G+ neutrophils, (D) CD45+ Ly6G–CD64low Ly6C+ monocytes, (E) CD45+ Ly6G–CD64+ CD11c+ alveolar macrophages, (F) CD45+ Ly6G–CD64+ CD11b+ interstitial macrophages. Representative dot plots are shown. [file Image_1.JPEG]
